# Gastrodin protects against chronic inflammatory pain by inhibiting spinal synaptic potentiation

**DOI:** 10.1038/srep37251

**Published:** 2016-11-17

**Authors:** Mei-Mei Xiao, Yu-Qi Zhang, Wen-Ting Wang, Wen-Juan Han, Zhen Lin, Rou-Gang Xie, Zhi Cao, Na Lu, San-Jue Hu, Sheng-Xi Wu, Hui Dong, Ceng Luo

**Affiliations:** 1 Department of Anesthesiology, Xijing Hospital, Fourth Military Medical University, Xi’an, 710032, China; 2 Department of Neurobiology and Collaborative Innovation Center for Brain Science, Fourth Military Medical University, Xi’an, 710032, China; 3 Department of Anesthesiology, Weifang People’s Hospital, 151 Guangwen Road, Weifang, 261041, China; 4 Class 2013, School of Clinical Medicine, Fourth Military Medical University, Xi’an 710032, China

## Abstract

Tissue injury is known to produce inflammation and pain. Synaptic potentiation between peripheral nociceptors and spinal lamina I neurons has been proposed to serve as a trigger for chronic inflammatory pain. Gastrodin is a main bioactive constituent of the traditional Chinese herbal medicine *Gastrodia elata Blume*, which has been widely used as an analgesic since ancient times. However, its underlying cellular mechanisms have remained elusive. The present study demonstrated for the first time that gastrodin exhibits an analgesic effect at the spinal level on spontaneous pain, mechanical and thermal pain hypersensitivity induced by peripheral inflammation, which is not dependent on opioid receptors and without tolerance. This analgesia by gastrodin is at least in part mediated by depressing spinal synaptic potentiation via blockade of acid-sensing ion channels. Further studies with miniature EPSCs and paired-pulse ratio analysis revealed the presynaptic origin of the action of gastrodin, which involves a decrease in transmitter release probability. In contrast, neither basal nociception nor basal synaptic transmission was altered. This study revealed a dramatic analgesic action of gastrodin on inflammatory pain and uncovered a novel spinal mechanism that could underlie the analgesia by gastrodin, pointing the way to a new analgesic for treating chronic inflammatory pain.

Inflammation of peripheral tissues causes spontaneous pain and hyperalgesia^1^. Although opioids and non-steroid anti-inflammatory drugs (NSAIDs) are widely used in the treatment of inflammatory pain, their long-term usage is complicated by side effects that seriously diminish the quality of life of patients, leading to poor compliance and rejection of therapy^2^,^3^,^4^. Increasing evidence has indicated that Chinese herbal medicine has emerged as an attractive alternative or supplement to current therapy for clinical pain^5^,^6^. Gastrodin (4-hydroxybenzyl alcohol 4-O-beta-D-glucopyranoside) (GAS) is the main bioactive constituent of the traditional Chinese herb “*Tian ma*” (*Gastrodia elata* Blume). Since ancient times in China, GAS has been widely used as an anticonvulsant, analgesic, and sedative^7^,^8^,^9^,^10^. As an analgesic, GAS is effective in relieving trigeminal neuralgia, migraine and vascular headache in clinical trials^11^,^12^. Despite these clinical observations, the scientific evaluation of GAS on different types of experimental pain is lacking, and the precise mechanisms underlying its analgesic action have remained unclear. Recently, our experimental study demonstrated that GAS attenuates diabetes-related neuropathic pain^13^. However, very little is known about exactly whether GAS exerts analgesia in inflammatory pain and which cellular and molecular mechanisms underlie this action.

Plasticity in peripheral nociceptors and their synapses with spinal neurons has been proposed as a cellular basis for the abnormal pain hypersensitivity following peripheral inflammation^1^,^14^,^15^,^16^,^17^,^18^. We and others recently reported that GAS was capable of eliminating nociceptor hyperexcitability via regulation of voltage- and ligand-gated ion channels, i.e. sodium and potassium channels as well as acid-sensing ion channels (ASICs)^13^,^19^. By virtue of housing the first crucial synapse in the pain pathway, the spinal dorsal horn, and especially the spinal lamina I, constitutes one of the most important sites for the integration of pain-related information^20^,^21^,^22^. Most lamina I neurons are nociceptive and are required for the full expression of hyperalgesia in various animal pain models^23^,^24^,^25^,^26^,^27^. Spinal synapses between nociceptor terminals and lamina I neurons undergo long-term potentiation (LTP) by conditioning stimulation and natural noxious stimulation, which is strongly implicated in inflammatory pain^14^,^28^,^29^,^30^,^31^. However, whether GAS exerts anti-nociceptive actions by suppressing spinal synaptic potentiation under inflammatory pain states has remained elusive. Substantial evidence has shown that ASIC channels display dramatic upregulation upon inflammation and are assumed to play a key role in inflammatory pain^32^,^33^,^34^,^35^. Recently, GAS has been shown to inhibit ASIC channels in DRG neurons^19^. We are therefore interested to know whether modulation of spinal synaptic potentiation by GAS is mediated by modulation of ASIC channels in the spinal cord.

By utilizing a combination of behavioral surveys, patch-clamp recordings and immunostaining methods, our study demonstrated that GAS inhibits spontaneous pain, mechanical and thermal hyperalgesia induced by peripheral inflammation, and this inhibition is not dependent on opioid receptors. This analgesia by GAS is at least in part mediated by its depression of spinal synaptic potentiation via inhibition of ASIC channels. Further studies with mEPSCs and PPR analysis revealed the presynaptic origin of GAS, involving a decrease in neurotransmitter release. Additionally, GAS was capable of depressing the hyperexcitability of postsynaptic spinal lamina I neurons under inflammatory states, and this inhibition was eliminated by the blockade of ASIC channels. This study revealed a significant analgesic action of GAS on inflammatory pain and uncovered a novel spinal mechanism that underlies the analgesia associated with GAS, pointing the way to a new analgesic for inflammatory pain.

## Results

### Systemic GAS inhibits persistent spontaneous pain-related behaviors in mice

**Figure 1:**
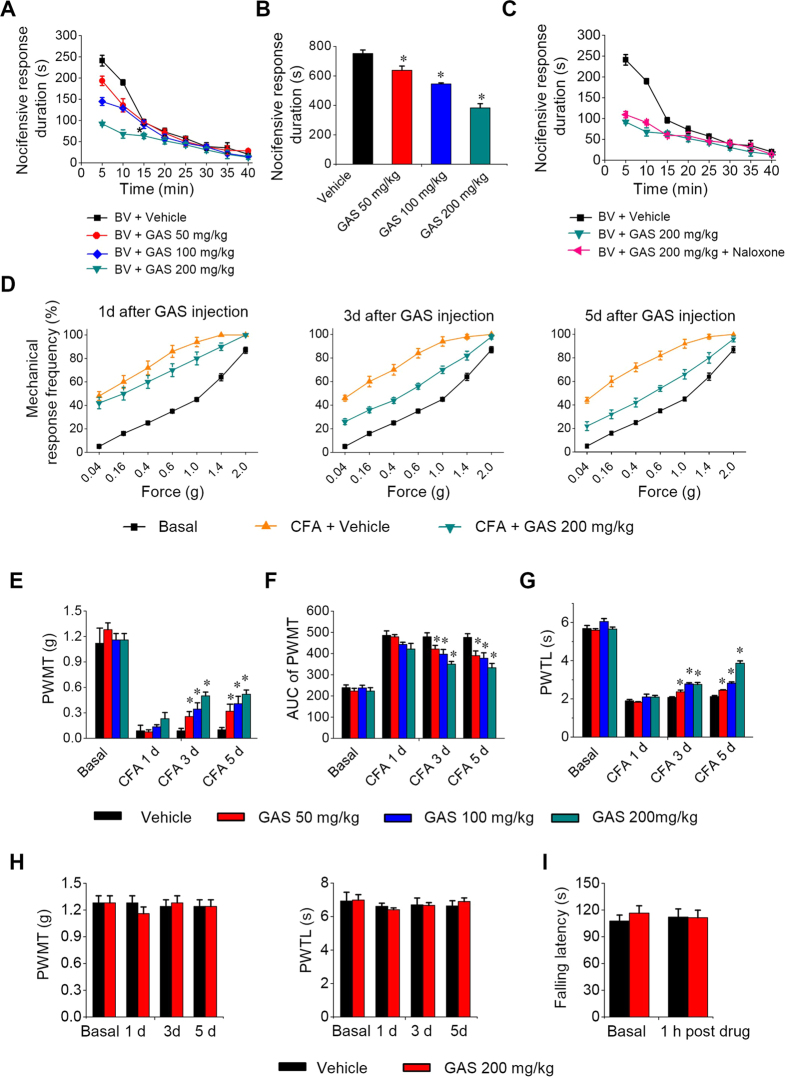
Intraperitoneal (i.p.) administration of GAS attenuated the development of spontaneous pain, mechanical and thermal pain hypersensitivity induced by paw inflammation. (**A**,**B**) The dose-dependent inhibitory effect of i.p. GAS (50, 100 and 200 mg/kg body weight) on the time course (**A**) and total response duration (**B**) of bee-venom-induced spontaneous pain (n = 8, *P* < 0.05). (**C**) The analgesic effect of GAS was not reversed by naloxone, an opioid receptor antagonist (n = 5, *P* > 0.05). (**D**) Representative traces showing the attenuation of CFA-induced mechanical pain hypersensitivity by i.p. GAS (200 mg/kg) at different time points after GAS administration. (E, F) Quantitative summary of the inhibition of i.p. GAS (50, 100 and 200 mg/kg) on the drop of paw withdrawal mechanical threshold (PWMT) (**E**), represented as integrated area under the curve, AUC, in (**F**) induced by CFA inflammation, indicating an analgesic effect on mechanical pain hypersensitivity (n = 8, *P* < 0.05). (**G**) Quantitative summary showing depression of thermal hyperalgesia by i.p. GAS (50, 100 and 200 mg/kg), manifesting as a prolonged paw withdrawal thermal latency (PWTL) to radiant heat stimuli compared to vehicle (n = 8, *P* < 0.05). (**H**,**I**) Neither basal nociception to mechanical and thermal stimuli (**H**) nor motor coordination (**I**) was altered by i.p. GAS (200 mg/kg) (n = 8, *P* > 0.05). All data are represented as mean ± S.E.M. *P < 0.05. PWMT, paw withdrawal mechanical threshold; PWTL, paw withdrawal thermal latency.

To investigate the effect of GAS on inflammatory pain, we first focused on a model of persistent inflammatory pain that is associated with spontaneous pain, namely unilateral hindpaw inflammation induced by the injection of bee venom^36^,^37^,^38^. The bee venom model is a well-established inflammatory pain model^36^,^38^,^39^,^40^. Intraplantar bee venom injection produced striking inflammation accompanied by a monophasic spontaneous nocifensive behavioral response characterized by flinching, lifting, licking or biting of the injected paw (Fig. 1A). This spontaneous pain lasted for 40 min and was most robust in the first 20 min. Intraperitoneal (i.p.) pretreatment with GAS (50, 100, 200 mg/kg body weight) twice daily for 3 days significantly inhibited spontaneous pain induced by bee venom in a dose-dependent manner (Fig. 1A). Compared to the vehicle group, the duration of spontaneous pain behaviors was reduced by 15.0%, 27.5%, and 49.2% upon GAS application at 50, 100, and 200 mg/kg, respectively (Fig. 1B, n = 8 for each dose, *P* < 0.05). These effects were not reversed by naloxone, an opioid receptor antagonist (Fig. 1C, n = 5). These results indicate that systemic GAS could relieve inflammation-induced spontaneous pain behaviors without dependence on opioid receptors.

### Systemic GAS attenuates mechanical allodynia and hyperalgesia as well as thermal hyperalgesia but not basal nociception in mice

We next examined the effect of GAS on a model of chronic inflammatory pain that is associated with mechanical allodynia and hyperalgesia, as well as thermal hyperalgesia. Following unilateral inflammation induced by intraplantar injection of Complete Freund’s Adjuvant (CFA), mice demonstrated the characteristic leftward and upward shift in the stimulus-response curve over basal curves, reflecting allodynia (measured here as responses to 0.04–0.6 g force, which are non-noxious in mice) as well as mechanical hyperalgesia (measured here as responses to 1.0–4 g force, which elicit a withdrawal behavior in mice) (Fig. 1D). At 24 h after CFA injection, when stable and significant hyperalgesia was produced, i.p. GAS or vehicle was administered twice daily for 5 days. Compared to the vehicle, i.p. administration of GAS (50–200 mg/kg) dramatically attenuated the development of mechanical allodynia and hyperalgesia in CFA-inflamed mice (Fig. 1D). A quantitative analysis showed that GAS increased the mechanical pain threshold dose- and time-dependently (Fig. 1D–F, n = 8, *P* < 0.05). In parallel, dramatic thermal hyperalgesia was observed at 24 h after CFA inflammation (Fig. 1G). I.p. GAS treatment (50–200 mg/kg) significantly alleviated the development of thermal hyperalgesia as well, manifesting as a prolonged response latency to radiant heat stimuli compared to the vehicle (Fig. 1G, n = 8, *P* < 0.05). Importantly, the analgesic efficiency of GAS was increased with a longer period of administration; therefore, no tolerance was observed. In striking contrast, neither basal nociception nor motor coordination was altered by GAS (200 mg/kg) (Fig. 1H, I, n = 8, *P* > 0.05).

### Intrathecal GAS suppresses mechanical allodynia and hyperalgesia as well as thermal hyperalgesia

**Figure 2:**
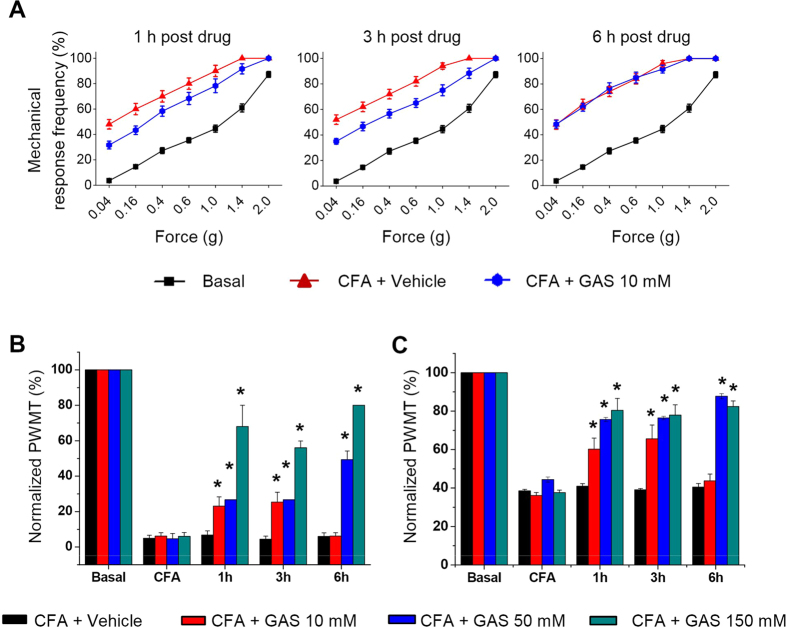
Intrathecal (i.t.) administration of GAS depressed mechanical and thermal pain hypersensitivity induced by unilateral CFA inflammation. (**A**) Representative traces showing the attenuation of CFA-induced mechanical pain hypersensitivity by a single bolus of i.t. GAS (10 mM) at different time points after GAS administration. (**B**) Quantitative analysis showing that i.t. GAS increased the response threshold to mechanical stimuli compared to vehicle treatment (n = 8, *P* < 0.05). (**C**) Quantitative summary showing the depression of thermal hyperalgesia by i.t. GAS (n = 8, *P* < 0.05). All data are represented as mean ± S.E.M. *P < 0.05.

To address whether GAS exerted an analgesic action at the spinal level, intrathecal (i.t.) GAS (10, 50, 150 mM) was applied at 24 h after CFA inflammation. Compared to the vehicle-treated group, a single bolus of i.t. GAS resulted in a dose-dependent increase of the mechanical threshold to von Frey hairs (Fig. 2A,B, n = 8, *P* < 0.05) and thermal latency to radiant heat stimulation (Fig. 2C, n = 8, *P* < 0.05), indicating attenuation of mechanical allodynia and hyperalgesia as well as thermal hyperalgesia. This anti-nociceptive effect of GAS was evident 1 h and 3 h after drug delivery but decayed from 6 h afterwards at 50 mM. At higher concentrations of GAS, it exerted strong anti-nociception at all times points tested. This result extends our previous finding that apart from the periphery^35^, GAS exerted obvious analgesia at the spinal level.

### GAS inhibits spinal c-Fos expression induced by inflammation

**Figure 3:**
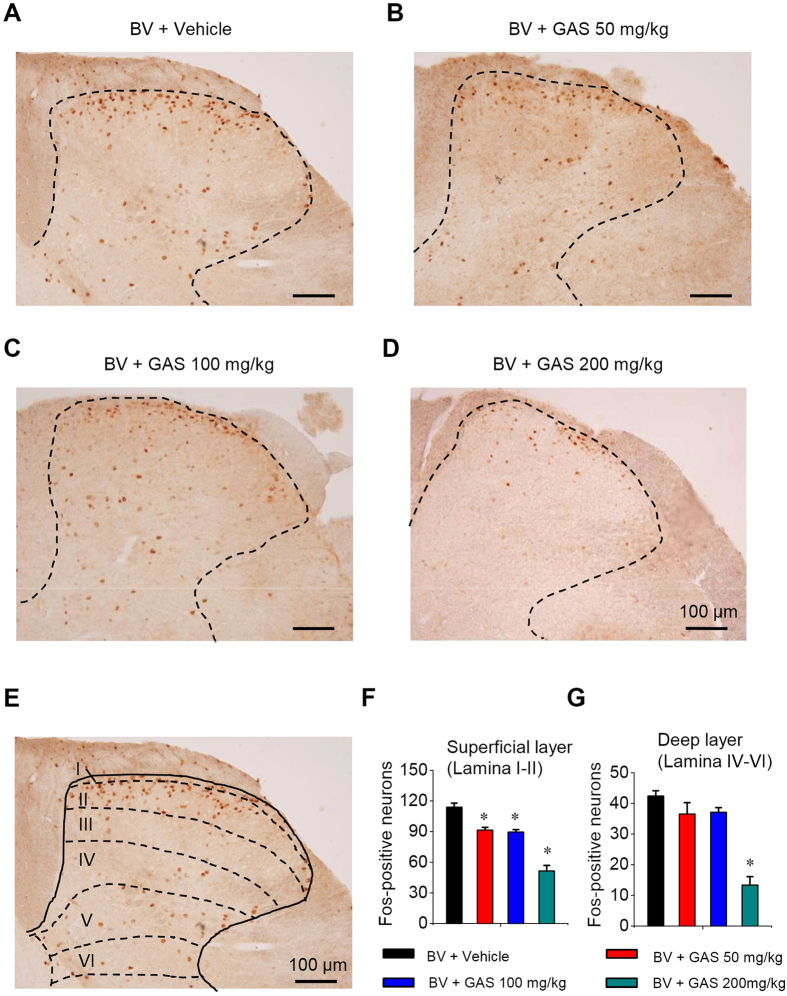
GAS inhibited spinal c-Fos expression induced by intraplantar bee venom injection. (**A**–**D**) Typical examples of the induction of c-Fos protein in the spinal dorsal horn induced by bee venom injection in vehicle-treated mice (**A**) and in groups treated with different doses of GAS (**B**–**D**). (**E**) Schematic diagram showing the counting of c-Fos-positive cells in different layers of the spinal dorsal horn. (**F**,**G**) Quantitative summary showing the inhibition of c-Fos expression in both the superficial (**F**) and deep layer of the spinal dorsal horn by GAS in a dose-dependent manner (n = 3, *P* < 0.05). All data are represented as mean ± S.E.M. Scale bar represents 100 μm in (**A**–**E**). *P < 0.05.

To obtain an overview of the spinal cord that contributes to the analgesia induced by GAS, we sought a readout that permits the assessment of populated neuronal activity by a functional marker. The proto-oncogene immediate early gene c-fos and its product, c-Fos protein, are very useful markers for monitoring neuronal activity in the pain pathways temporally and spatially^36^,^41^,^42^. Consistent with previous reports^36^, hind-limb intraplantar injection of bee venom produced a significant upregulation of c-Fos in the spinal dorsal horn of L4-5 segments (Fig. 3A). Dense c-Fos-immunoreactivity (c-Fos-ir) was seen in the superficial (lamina I-II) and deep (lamina IV-V) lamina, where the nociceptive primary afferents mainly terminate (Fig. 3A). Compared to the vehicle, systemic delivery of GAS (50–200 mg/kg) dose-dependently depressed c-Fos expression in both superficial and deep lamina of the spinal dorsal horn (see Fig. 3B–D for typical examples). The mean number of c-Fos-ir cells per section in the L4-5 dorsal horn superficial and deep lamina of the vehicle group was 113.89 ± 4.04 and 42.38 ± 1.74, respectively (Fig. 3F,G, spinal lamina differentiation was depicted in Fig. 3E). Following GAS administration, the mean number of c-Fos-ir cells in two regions was reduced by 19.69 ± 2.37% and 12.49 ± 3.63% at a dose of 50 mg/kg, 21.40 ± 2.19% and 13.89 ± 8.86% at 100 mg/kg, and 54.85 ± 4.79% and 68.82 ± 6.23% at 200 mg/kg, respectively (Fig. 3E,F). These results indicate that GAS possesses the ability to inhibit increased neuronal activity of spinal nociceptive neurons in inflammatory pain states.

### GAS inhibits C-fiber evoked EPSCs (c-eEPSCs) in spinal lamina I neurons

**Figure 4:**
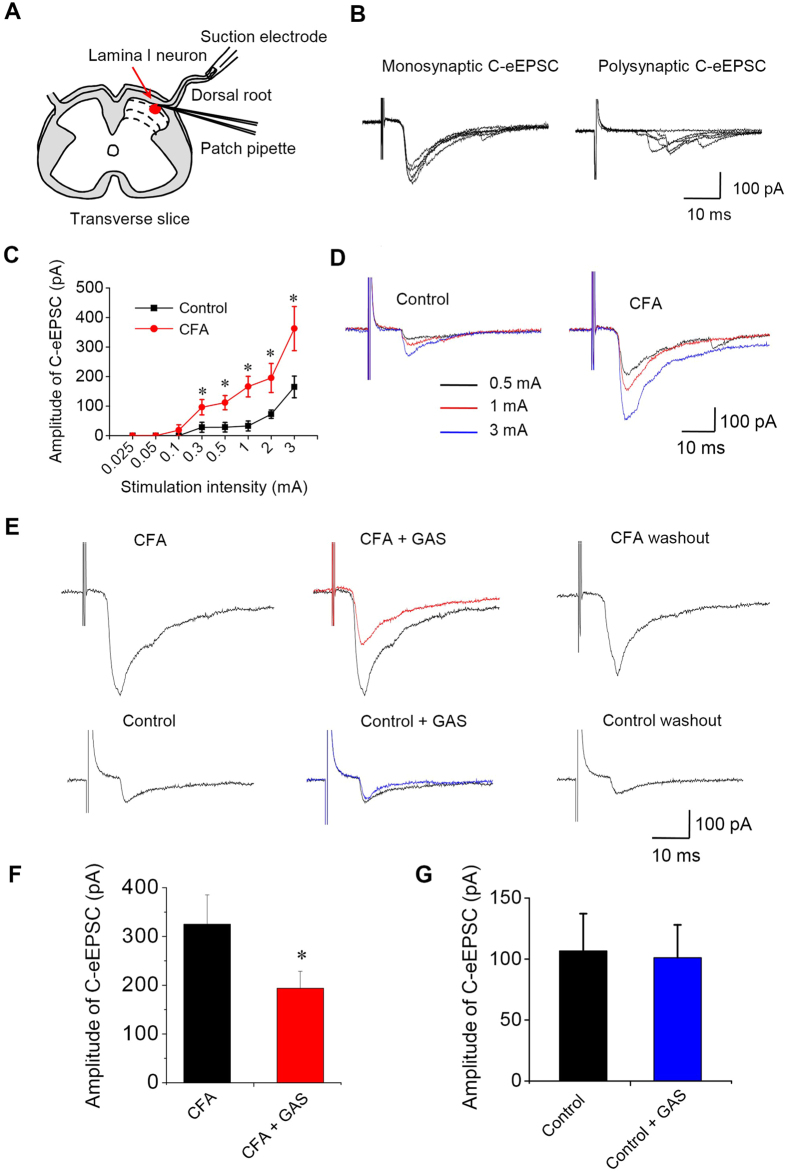
GAS preferentially inhibited spinal synaptic potentiation under inflammatory pain states but with intact basal synaptic transmission. (**A**) Schematic diagram showing whole-cell patch-clamp recording from lamina I neurons in a spinal cord slice attached with a dorsal root. (**B**) Typical examples of monosynaptic C-eEPSCs and polysynaptic C-eEPSCs recorded in spinal lamina I neurons. (**C**) Input-output curves for synaptic transmission between C-fibers and spinal lamina I neurons in control and CFA-inflamed mice (n = 8, *P* < 0.05). (**D**) Typical traces of C-eEPSCs in response to increasing intensity of dorsal root stimulation from control and CFA-inflamed spinal slice. (**E**) Representative traces showing that bath application of GAS (300 μM) reversibly inhibited the peak amplitude of C-eEPSCs recorded from CFA-inflamed spinal slice (upper panels) but not that from control spinal slice (lower panels). Quantitative summary of the effect of GAS on C-eEPSC in CFA-inflamed and control mice are plotted in (**F**) (n = 10, *P* < 0.05) and (**G**) (n = 7, *P* > 0.05), respectively. All data are represented as mean ± S.E.M. *P < 0.05.

Plasticity in primary afferent synapses between peripheral nociceptors and spinal lamina I neurons can serve as a cellular basis for the development and maintenance of chronic pain following inflammation or nerve injury^14^,^31^. To explore the mechanisms by which GAS alleviates inflammatory pain at the spinal level, we further evaluated the effect of GAS on spinal synaptic transmission and potentiation. Whole-cell recordings were made from 15 spinal lamina I neurons in 14 mice (the experimental scheme is shown in Fig. 4A). We recorded monosynaptic C-fiber-evoked EPSCs (C-eEPSCs) at a *V*_H_ of −70 mV in the presence of gabazine (10 μM), strychnine (1 μM) and AP-5 (50 μM), which are antagonists of GABA_A_ receptors, glycine receptors and NMDA receptors, respectively (Fig. 4A,B). Polysynaptic C-eEPSCs were excluded from further experiments. To determine pain-related plastic changes of excitatory synaptic transmission, input-output (I-O) relationships were obtained by measuring peak amplitudes of C-eEPSCs as a function of afferent fiber stimulus intensity for each neuron derived from control and CFA-inflamed mice. Compared with the controls (n = 8 neurons/5 mice), the I-O curve of C-eEPSCs shifted leftward and upward significantly after CFA inflammation (see Fig. 4C for quantitative analysis and Fig. 4D for typical examples, n = 8 neurons/4 mice, *P* < 0.05). Analysis of miniature ESPCs (mEPSCs) revealed that upon CFA inflammation, the frequency but not the amplitude of mEPSCs was markedly increased (see Supplementary Fig. 1A for typical examples and B for quantitative analysis, n = 8 neurons/4 mice, *P* < 0.05). These results suggest that synaptic transmission of primary afferent synapses underwent a plastic potentiation in the spinal cord after natural peripheral inflammation, which is consistent with what was reported by the conditioning stimulus in previous studies^14^,^28^,^29^,^30^.

In 83.3% (15 of 18) of spinal lamina I neurons examined, superfusing GAS (300 μM) significantly depressed the peak amplitude of C-eEPSCs recorded from CFA-inflamed mice in a reversible manner (Fig. 4E, upper panels). A quantitative analysis revealed an average inhibition of 42.8% of C-eEPSC by GAS (Fig. 4F, n = 15 neurons/10 mice, *P* < 0.05). In striking contrast, GAS did not alter the basal synaptic transmission from control mice (Fig. 4E, lower panels). As shown in Fig. 4G, the peak amplitude of C-eEPSC was not significantly changed after superfusion of GAS in control mice (Fig. 4G, n = 7 neurons/5 mice, *P* > 0.05). We can infer from the above that GAS preferentially inhibits synaptic potentiation occurring in inflammatory pain states, with intact basal synaptic transmission.

### Inhibitory action of GAS on spinal synaptic potentiation is presynaptic in origin

#### Effects of GAS on mEPSCs

**Figure 5:**
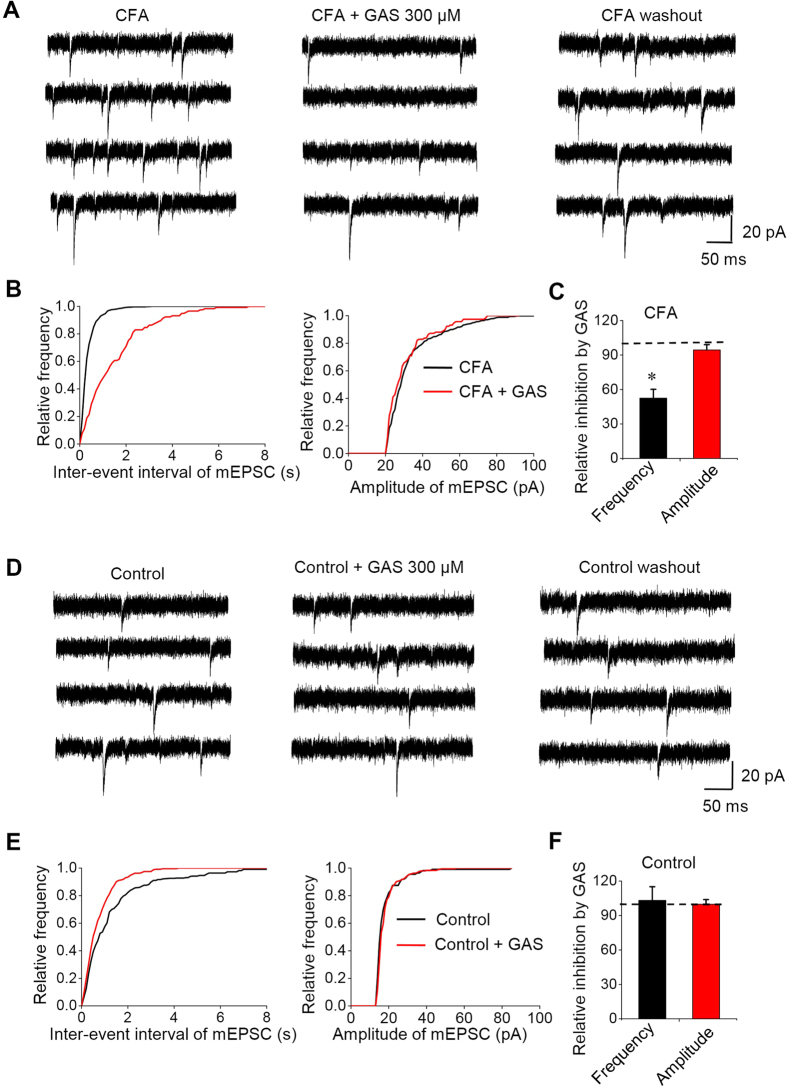
GAS-induced inhibition of mEPSCs recorded from CFA-inflamed mice but not control mice. (**A**) Four consecutive mEPSCs traces before (left), during the action of GAS (300 μM) (middle), and after the washout of GAS (right) from the CFA-inflamed slice. (**B**) Cumulative distributions of the inter-event interval (left) (n = 7, *P* < 0.05) and amplitude (right) (n = 7, *P* > 0.05) of mEPSCs before (black line) and during (red line) the action of GAS on the CFA-inflamed slice. (**C**) Quantitative summary showing the inhibitory rate of GAS on the frequency (n = 7, *P* < 0.05) and amplitude (n = 7, *P* > 0.05) of mEPSCs from the CFA-inflamed slice. (**D**) Four consecutive mEPSCs traces before (left), during the action of GAS (300 μM) (middle), and after washout of GAS (right) from the control slice. (**E**) Cumulative distributions of the inter-event interval (left) and amplitude (right) of mEPSCs before (black line) and during (red line) the action of GAS on the control slice (n = 7, *P* > 0.05). (**F**) Quantitative summary showing the effect of GAS on the frequency and amplitude of mEPSCs from control slice (n = 7, *P* > 0.05). All data are represented as mean ± S.E.M. *P < 0.05.

As illustrated in Fig. 5A–C, superfusing GAS (300 μM) resulted in a reversible reduction in mEPSCs frequency recorded in spinal lamina I neurons from CFA-inflamed mice (52.5 ± 7.66% of control following GAS application, n = 7 neurons/4 mice, *P* < 0.05; representative traces are shown in Fig. 5A and quantitative analysis shown in Fig. 5C). In contrast, mEPSCs amplitude did not change significantly in response to GAS (94.3 ± 4.7% of control, *P* > 0.05). Figure 5B shows the actions of GAS on the cumulative distributions of the inter-event interval and amplitude of mEPSCs (Fig. 5B). Whereas GAS increased the proportion of mEPSCs with a longer inter-event interval, it had no effect on the cumulative distribution of mEPSCs amplitude, indicating a presynaptic origin of action. Unlike the dramatic effect on mEPSCs from CFA-inflamed mice, GAS did not significantly change either the frequency or amplitude of mEPSCs from control mice (n = 7 neurons/4 mice, *P* > 0.05) (Fig. 5D–F).

#### Effects of GAS on paired-pulse ratio of C-eEPSCs

**Figure 6:**
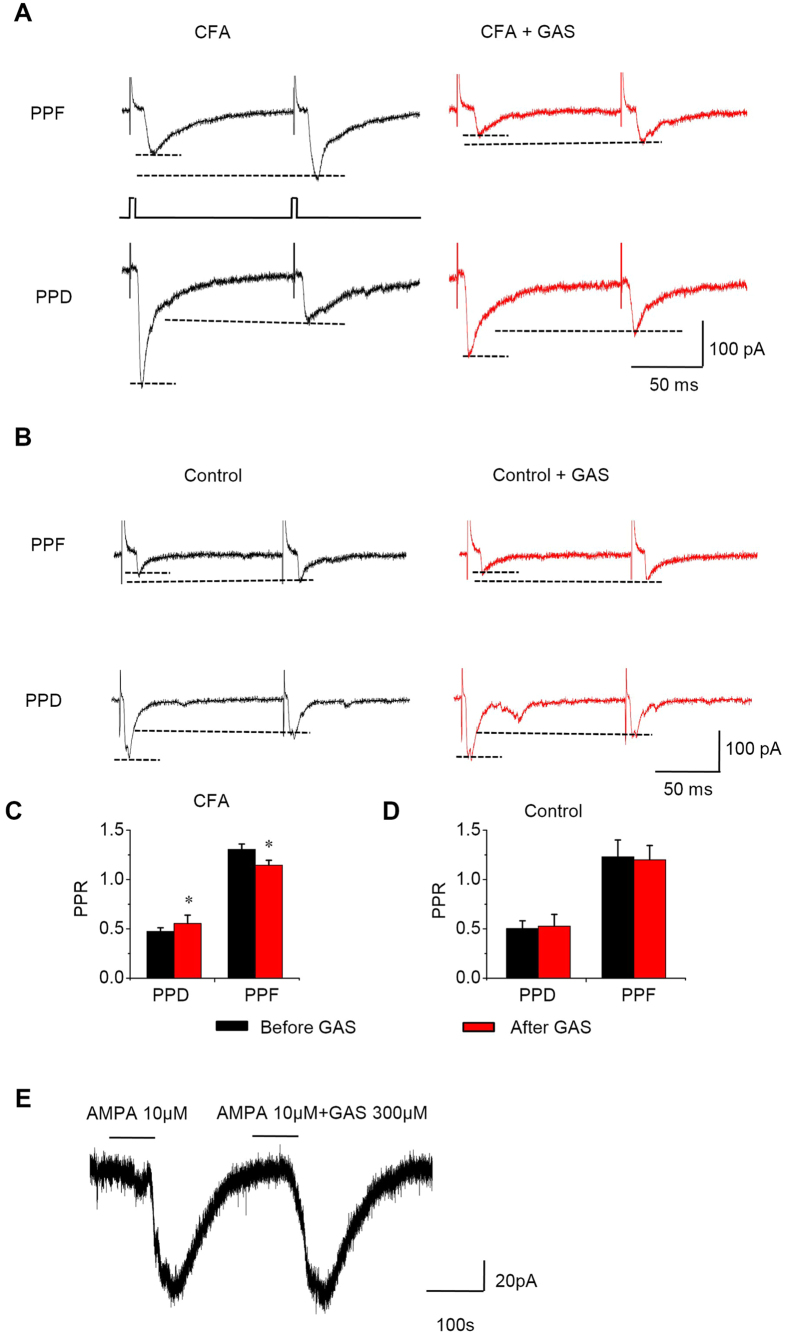
Analysis of the action of GAS on the paired-pulse facilitation (PPF) and paired-pulse depression (PPD) of C-eEPSCs induced by pairs of stimuli at an inter-stimulus interval of 110 ms as well as AMPA response in spinal lamina I neurons from control and CFA-inflamed mice. (**A**,**B**) Traces of typical recordings showing PPF or PPD prior to (left, black) and during GAS application (right, red) from CFA-inflamed (**A**) and control mice (**B**). (**C**,**D**) Quantitative analysis of the paired-pulse ratio prior to and during GAS action from CFA-inflamed (**C**) and control mice (**D**). Note that the PPR displayed a significant change following GAS application in CFA-inflamed mice (n = 10, *P* < 0.05), but this change did not occur in control mice (n = 10, *P* > 0.05). (**E**) The effect of GAS (300 μM) on the response of lamina I neurons from CFA-inflamed mice (n = 4) to AMPA (10 μM) application (*P* > 0.05). The horizontal bars shown above the record indicate the period of time during which the drugs were applied. All data are represented as mean ± S.E.M. *P < 0.05.

To further consolidate the presynaptic mechanisms of GAS, we focused on the analysis of C-eEPSCs elicited by a paired-pulse stimulation, which represents a short-lasting change in the second evoked EPSC when it follows shortly after the first and is well accepted as an indication of presynaptic mechanisms [50]. In spinal slices derived from CFA-inflamed mice, we found evidence of paired-pulse facilitation (PPF) as well as paired-pulse depression (PPD) of C-eEPSCs prior to GAS treatment (typical traces are shown in Fig. 6A,B). The analysis of paired-pulse ratio (PPR) of C-eEPSCs revealed that GAS (300 μM) led to a significant change in PPR (see typical examples in Fig. 6A and quantitative analysis in Fig. 6C, n = 10 neurons/7 mice, *P* < 0.05). In striking contrast, this significant change in PPR was not observed in control mice (see typical examples in Fig. 6B and quantitative analysis in Fig. 6D, n = 10 neurons/7 mice, *P* > 0.05). In conclusion, the mEPSCs and PPR analyses strongly support the inference that the inhibition of synaptic potentiation by GAS under inflammatory states comes about via presynaptic mechanisms involving a decrease in release probability.

#### Effects of GAS on AMPA-induced response

To determine whether there is any effect of GAS on the sensitivity of spinal neurons to L-glutamate, we examined whether an AMPA response (10 μM) was affected by GAS (300 μM). As seen in Fig. 6E, GAS did not alter the peak amplitude of the AMPA response (98.54 ± 1.71% of control, n = 4 neurons/3 mice, *P* > 0.05). This finding indicates that GAS did not exert significant effects on the responsiveness of postsynaptic AMPA receptors.

### Effect of GAS on the membrane properties and hyperexcitability of spinal lamina I neurons in inflamed states

**Figure 7:**
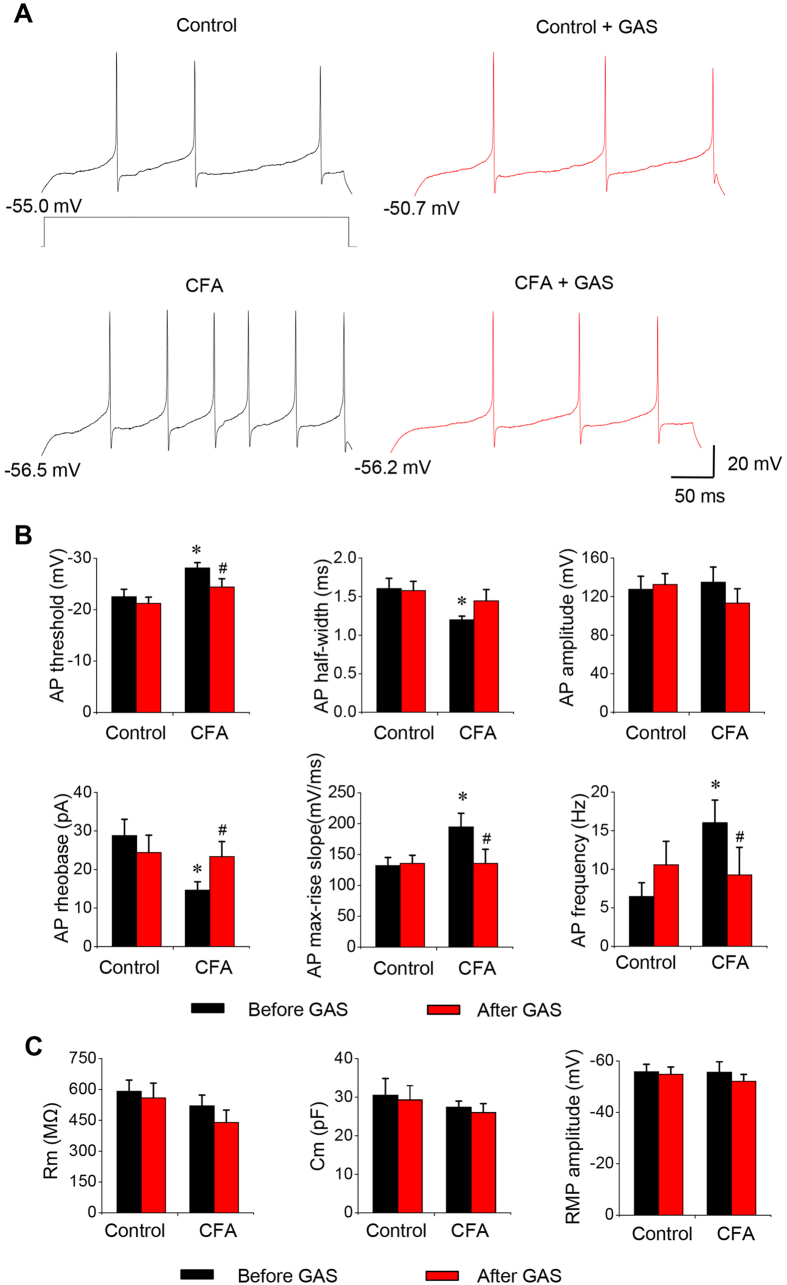
The inhibitory effect of GAS on the membrane properties and hyperexcitability of spinal lamina I neurons from CFA-inflamed mice. (**A**) Representative traces showing the spike firings to a depolarizing current step prior to (left panels) and during GAS application (right panels) in spinal lamina I neurons from control (upper panels) and CFA-inflamed (lower panels) mice. Note that the traces before and after GAS application in each group were from the same neuron. (**B**) Quantitative analysis showing the active membrane properties, such as AP threshold, amplitude, half-width, rheobase, max-rise slope and firing frequency in spinal lamina I neurons from control and CFA-inflamed mice prior to (black column) and during GAS application (red column). Note that lamina I neurons from CFA-inflamed mice displayed lowered AP thresholds, shortened AP half-widths, reduced rheobase, increased AP rise slope and increased firing frequency compared to controls. These changes in CFA-inflamed mice were normalized by GAS. In contrast, GAS exerted no effect on the membrane properties of lamina I neurons from control mice. (**C**) Passive membrane properties of lamina I neurons from control and CFA-inflamed mice as well as the action of GAS on these passive membrane properties. All data are represented as mean ± S.E.M. *P < 0.05 compared to the control mice. ^#^*P* < 0.05 compared to prior to GAS application in CFA-inflamed mice.

Given the strong inhibition of primary afferent synaptic potentiation by GAS in inflamed mice, we further tested the net effect of GAS on the membrane properties and excitability of postsynaptic lamina I neurons, which are the output of the primary afferent synapse. Following CFA inflammation, spinal lamina I neurons exhibited augmented excitability, which manifested as a lowered AP threshold, shortened AP half-width, reduced rheobase and increased AP rise slope (Fig. 7A,B, n = 16 neurons/10 inflamed mice *vs* n = 16 neurons/8 control mice, *P* < 0.05). In contrast, the passive membrane properties of spinal lamina I neurons were not different between the control and CFA groups (Fig. 7C, n = 16 neurons/10 inflamed mice *vs* n = 16 neurons/8 control mice, *P* > 0.05). Bath application of GAS (300 μM) significantly decreased the excitability of spinal lamina I neurons recorded from CFA-inflamed mice (Fig. 7A,B, n = 10 neurons/8 mice, *P* < 0.05). For example, AP half-width, AP rheobase, AP slope and spike threshold in inflamed lamina I neurons was normalized by GAS (Fig. 7B, n = 10 neurons/8 inflamed mice, *P* < 0.05). More importantly, the mean firing frequency induced by a depolarizing current step was much higher in spinal lamina I neurons from inflamed mice than that from controls (Fig. 7A,B, n = 16 neurons/10 inflamed mice *vs* n = 16 neurons/8 control mice, *P* < 0.05). This elevated firing frequency was markedly reduced by application of GAS (300 μM) (Fig. 7A,B, n = 10 neurons/8 inflamed mice, *P* < 0.05). The passive membrane properties of inflamed lamina I neurons were, however, not altered by GAS (Fig. 7C, n = 10 neurons/8 inflamed mice, *P* > 0.05). In striking contrast to the dramatic effect of GAS on inflamed lamina I neurons, the same concentrations of GAS did not exert obvious effects on the active and passive membrane properties, as well as firing frequency to depolarizing currents, in control lamina I neurons (Fig. 7A,C, n = 10 neurons/8 mice, *P* > 0.05).

**Figure 8:**
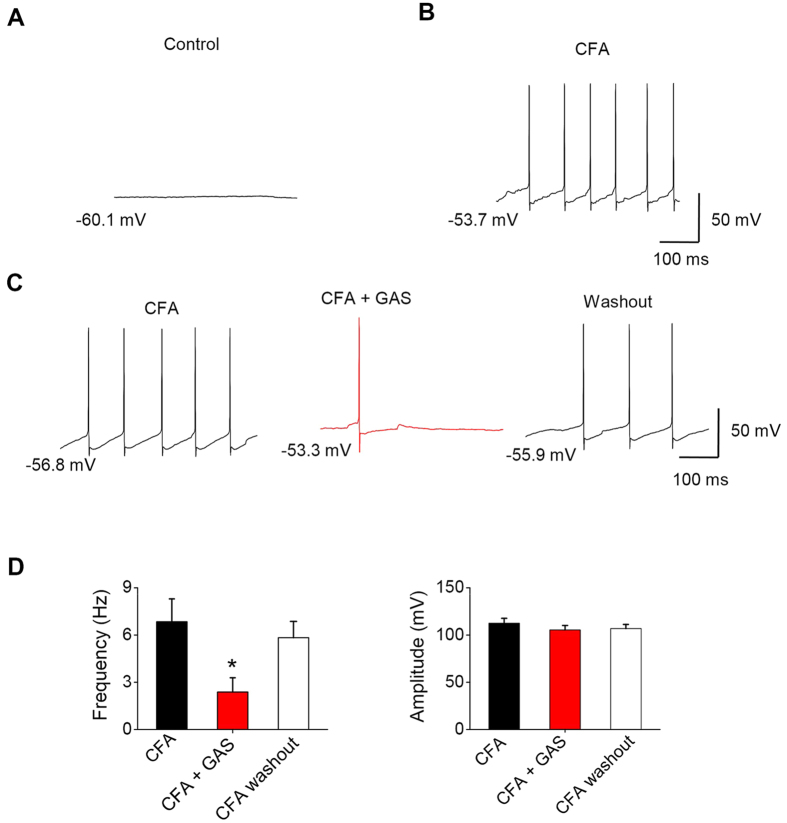
The inhibitory action of GAS on spontaneous firing in spinal lamina I neurons induced by CFA inflammation. (**A**,**B**) Typical traces showing spontaneous firing in spinal lamina I neurons from control (**A**) and CFA-inflamed mice (**B**). (**C**) Representative recordings of spontaneous firing prior to (left), during the action of GAS (middle) and washout of GAS in the same lamina I neurons. (**D**) Quantitative summary showing the effect of GAS on the frequency (left, n = 7, *P* < 0.05) and amplitude (right, n = 7, *P* > 0.05) of spontaneous firing in lamina I neurons. All data are represented as mean ± S.E.M. *P < 0.05.

Previous studies have reported that spinal dorsal horn neurons displayed spontaneous firing with extracellular single unit recording under inflammatory states^38^. In the present study, spontaneous firing was seen in 28% (7/28) of spinal lamina I neurons under whole-cell current-clamp recording upon testing at 24 h after CFA inflammation, which was not seen in the control mice (Fig. 8A,B). The frequency of spontaneous firing in CFA-inflamed mice reached 6.84 ± 1.46 Hz (Fig. 8C). Superfusion of GAS (300 μM) almost eliminated the frequency of spontaneous firing (Fig. 8B,C). The inhibitory rate averaged 65.2% by GAS (Fig. 8C, n = 7 neurons/4 mice, *P* < 0.05). Taken together, the above results suggest that GAS may protect against inflammatory pain via inhibition of spinal synaptic potentiation by reducing presynaptic transmitter release, further leading to the reduction of hyperexcitability of spinal lamina I neurons. However, the reduction of hyperexcitability of spinal lamina I neurons by GAS was not excluded by direct regulation of spinal neurons. This finding was supported by our recent study showing that GAS eliminated the hyperexcitability of nociceptive DRG neurons in diabetic rats by decreasing sodium currents and increasing potassium currents^13^.

### Inhibitory actions of GAS on spinal synaptic potentiation and neuronal hyperexcitability are mediated by ASIC channels

**Figure 9:**
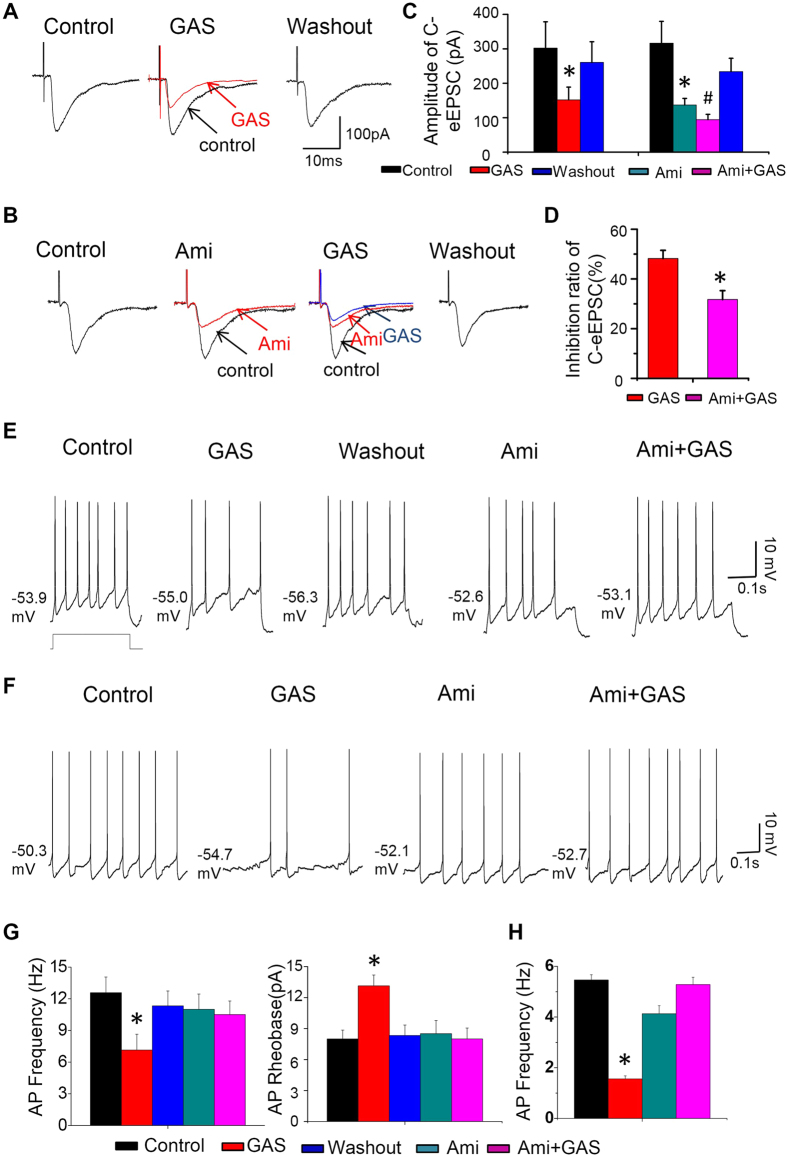
GAS-induced inhibition of C-eEPSCs and spinal neuronal hyperexcitability in CFA-inflamed mice was mediated by blockade of ASIC channels. (**A**,**B**) Typical traces showing the action of GAS (300 μM) on C-eEPSCs in the absence (**A**) and presence of a nonselective antagonist of ASICs, amiloride (200 μM) (**B**). Quantitative summaries are plotted in (**C**,**D**) (n = 5, *P* < 0.05), respectively. (**E**) Representative traces showing that GAS-induced inhibition of spinal neuronal hyperexcitability in response to depolarizing current injection (**F**) and spontaneous firing in CFA-inflamed mice were significantly suppressed by the blockade of ASICs (n = 8, *P* < 0.05). (**G**) Quantitative analysis showing that GAS-induced inhibition of firing frequency (left) and GAS-induced increase of rheobase in CFA-inflamed mice was blocked by amiloride. (**H**) Quantitative analysis showing that GAS-induced inhibition of spontaneous firing was eliminated by amiloride. All data are represented as mean ± S.E.M. *P < 0.05.

Acid-sensing ion channels (ASICs) have been assumed to play a key role in inflammatory pain^32^,^33^,^34^,^35^. A recent study has reported that GAS was able to inhibit the activity of ASICs in rat primary sensory neurons^19^. Therefore, we are interested to know whether ASICs are involved in the inhibitory effect of GAS on spinal synaptic potentiation under inflammatory states. As shown in Fig. 9, when examined in the presence of the nonselective antagonist of ASIC channels, amiloride (200 μM), GAS-induced inhibition of C-eEPSCs in CFA-inflamed mice was mostly eliminated (Fig. 9A–C, n = 5 neurons/4 mice, *P* < 0.05), suggesting that inhibition of spinal synaptic potentiation by GAS may be partially mediated by ASIC channels. Here, amiloride by itself significantly suppressed the amplitude of C-eEPSCs (Fig. 9B,C, n = 5 neurons/4 mice, *P* < 0.05), indicative of the involvement of ASIC channels in spinal synaptic potentiation. Additionally, in examining the neuronal hyperexcitability of spinal lamina I neurons in CFA-inflamed mice, co-application of amiloride and GAS greatly reduced the inhibitory effect of GAS on firing frequency induced by depolarizing current injection (Fig. 9E,G, n = 8 neurons/5 mice). In parallel, GAS-induced inhibition of spontaneous firing in spinal lamina I neurons was also alleviated by a blockade of ASIC channels (Fig. 9F,H, n = 6 neurons/6 mice).

## Discussion

In contrast to the wide implication as an analgesic in clinic trials, very few studies have addressed the cellular and molecular mechanisms underlying analgesic actions of GAS. In the present study, by utilizing a combination of behavioral surveys and electrophysiological recordings, as well as immunostaining, we demonstrated, for the first time, the spinal analgesic action of gastrodin in inflammatory pain. Gastrodin exerts this analgesic effect by inhibiting spinal synaptic potentiation between primary afferent fibers and spinal lamina I neurons in inflammatory states; this inhibition was partially mediated by ASIC channels and is presynaptic in origin.

### Gastrodin inhibits inflammation-induced pain hypersensitivity in mice

Although opioids and NSAIDs constitute the current mainstay of treatment of clinical pain, long-term use of these drugs causes unwanted side effects which limit their broader application^2^,^3^,^4^. In recent years, Chinese herbal medicine has emerged as an alternative and complementary approach to current therapy to relieve patients’ pain^5^,^8^. Among them, gastrodin, a main constituent of the traditional Chinese herb *Tianma (Gastrodia elata Blume*) has been reported to be effective in relieving trigeminal neuralgia, migraine and vascular headache in clinical trials in China^11^,^12^. However, none of the experimental data systemically examining the role of GAS on animal pain models and its underlying cellular mechanisms have been reported. Until recently, our group demonstrated that gastrodin was able to alleviate neuropathic pain associated with diabetes mellitus by reducing the hyperexcitability of peripheral nociceptors. Apart from neuropathic pain, inflammatory pain is seen frequently in the clinic and is usually caused by tissue damage/inflammation. Whether and by which mechanisms gastrodin can protect against inflammatory pain has remained elusive.

To address this question, we utilized two inflammatory models, namely unilateral intraplantar injection of bee venom and CFA in mice, which are well studied to induce spontaneous nociception and hyperalgesia as well as allodynia^30^,^38^. One of the most striking findings of the present study is that systemic administration of gastrodin effectively attenuated bee venom-induced spontaneous pain and CFA-induced mechanical and thermal pain hypersensitivity. A further observation with intrathecal administration revealed that spinal dorsal horn plays a pivotal role in analgesia associated with gastrodin. In striking contrast, gastrodin did not alter basal nociception and motor coordination. More importantly, the analgesic action of gastrodin was not dependent on opioid receptors. Repeated gastrodin treatment resulted in increasing analgesic efficacy without inducing tolerance. It can be inferred from these results that gastrodin may selectively abolish pathological pain without affecting basal nociception, pointing the way to a new analgesic for the treatment of pathological pain.

### Gastrodin depresses increased spinal neuronal activity in inflammatory states

Numerous studies have consistently shown that injury or inflammation produces increased neuronal excitability in the regions that are involved in the processing of painful information from the periphery to the CNS^1^,^16^,^18^,^43^. The spinal dorsal horn neurons receive major projections from nociceptive DRG neurons and ascend incoming pain signals to the cortex via several ascending pathways so that pain is ultimately perceived in its multiple dimensions. Several lines of evidence have suggested that c-Fos may be useful as a sensitive marker of the neuronal activation and plasticity following noxious stimuli^36^,^41^,^42^. Following inflammation by intraplantar bee venom stimuli, a dramatic increase of c-Fos induction was seen in the spinal dorsal horn, especially in superficial (lamina I and II) and deep (lamina IV/V) lamina, indicating increased neuronal activity of spinal dorsal horn after inflammation. This result is generally consistent with previous reports by Luo *et al*.^36^. Administration of gastrodin yielded a significant suppression of c-Fos upregulation in the spinal dorsal horn in a dose-dependent manner, indicating a key role of gastrodin on the increased neuronal activity of the spinal dorsal horn caused by peripheral inflammation.

### Gastrodin-induced analgesia is mediated by the inhibition of spinal synaptic potentiation and neuronal hyperexcitability of spinal lamina I neurons via blockade of ASIC channels

Spinal lamina I neurons receive major projections of nociceptive primary afferent fibers and play a crucial role in the development of pain and hyperalgesia^20^,^21^,^22^,^24^,^25^,^26^. Converging lines of evidence have indicated that primary afferent C-fibers-evoked EPSCs (C-eEPSCs) in spinal lamina I neurons are susceptible to LTP by electrical conditioning stimulus of the dorsal root, which mimics repetitive firings induced by inflammation or injury^28^,^29^,^30^. This LTP is assumed to be implicated as a cellular basis for the development and maintenance of chronic pain following inflammation or injury^14^,^31^. Despite wide acceptance of LTP induced by artificial electrical conditioning stimulus, whether C-eEPSCs in spinal lamina I neurons displayed potentiation after natural chronic inflammation has remained elusive. To this end, the present study demonstrated that upon chronic inflammation by CFA, C-eEPSCs in spinal lamina I neurons exhibit marked potentiation in magnitude, manifesting as a leftward and upward shift of the I-O curve of C-eEPSCs in inflamed state over basal curve in normal state. This finding is inconsistent with a previous observation that CFA inflammation increases the incidence and magnitude of Aδ- but not C-eEPSCs in spinal lamina I neurons^44^. The obvious difference between our results and this study might be due to species difference (mice and rats), but the exact reason for this discrepancy is not known. Additionally, mEPSCs recorded in spinal lamina I neurons are increased in frequency by CFA inflammation. We can infer from the above that the strength of synaptic transmission at spinal nociceptive synapses undergoes plastic changes following inflammation, akin to a dial that can be turned up or down to regulate the levels of excitation in nociceptive pathways and accordingly tune the pain response of the organism.

Recently, we reported that gastrodin produced a remarkable inhibition in C-nociceptor hyperexcitability in diabetic rats, by which relief of diabetic neuropathic pain was generated^13^. It is well accepted that inflammation or injury-induced nociceptor hyperexcitability may drive enhanced transmitter release, leading to synaptic potentiation in the spinal cord and further along the ascending pain pathways, which in turn results in the exaggerated pain sensitivity after inflammation or injury^14^,^16^,^18^. Therefore, we expected that gastrodin may play a role in synaptic potentiation in the inflamed state. Another major finding of the present study is that gastrodin dramatically attenuated the magnitude of C-eEPSCs in CFA-inflamed spinal slices. In striking contrast, no change in C-eEPSCs in control spinal slices was observed in response to gastrodin. A further observation demonstrated that GAS-induced inhibition of spinal synaptic potentiation was mostly eliminated in the presence of an ASIC channel antagonist. This result is supported by a recent report that GAS possesses the ability to suppress the activity of ASIC channels in primary sensory neurons^19^. The above results strongly suggest that gastrodin may effectively and selectively suppress synaptic potentiation at synapses between nociceptive C afferents and spinal lamina I neurons via blockade of ASIC channels, without affecting basal synaptic transmission in the normal state. This notion may explain the selective analgesic action of gastrodin in inflammatory pain hypersensitivity but not in basal nociception.

Synaptic potentiation in the spinal level under pathological states can come about presynaptically or post-synaptically^14^. Thus, pre- and post-synaptic contributions to gastrodin-induced inhibition of synaptic potentiation have not been worked out so far. To address this question, we performed two lines of assays, including mEPSCs and a PPR analysis. The present study demonstrated for the first time that gastrodin application significantly reduced the frequency, but not the amplitude, of mEPSCs recorded in CFA-inflamed spinal slices. The PPR analysis revealed that the depression of C-eEPSCs by gastrodin in CFA-inflamed spinal slices was associated with an obvious change in the PPR. Given the assumption that frequency of mEPSCs and change in PPR represent an indication of presynaptic mechanism (i.e., a change in release probability)^45^, the above results support the inference that the inhibition of synaptic potentiation by gastrodin may come about via presynaptic mechanisms involving a decrease in release probability. This decreased transmitter release probability caused by GAS could lead to reduced inflow of painful signals to the spinal cord, leading to reduced hyperexcitability of spinal lamina I neurons, which in turn results in behavioral analgesia under inflammatory states. This notion is supported by the fact that GAS inhibited the increased hyperexcitability of spinal lamina I neurons and the pain hypersensitivity associated with tissue inflammation.

In summary, the present study systematically demonstrated for the first time the spinal anti-nociceptive action of GAS in inflammatory pain and revealed a cellular basis for this anti-nociception through a presynaptic mechanism in the spinal cord. This finding points the way to a new analgesic for the treatment of pathological pain, including inflammatory pain.

## Materials and Methods

### Animals and behavioral testing

#### Animals

All animal use procedures were approved by the Institutional Animal Use and Protection Committee, Fourth Military Medical University. All the testing was carried out in accordance with the approved guidelines. All behavioral measurements were done in awake, unrestrained adult C57BL6 mice. The animals were housed in plastic boxes at 22–26 °C with food and water ad libitum in the colony room. A 12:12 h light dark cycle with lights on at 08:00 was maintained and testing was done between 09:00 and 18:30. Mice were acclimatized to the laboratory and habituated to the experimental setups for at least 30 min each day for 5 days before testing.

#### Inflammatory pain models and behavioral tests

Inflammatory pain was induced by unilateral intraplantar injection of bee venom (0.2 mg/0.1 ml, 30 μl) or Complete Freund’s adjuvant (CFA; 30 μl) into mice hindpaws as described previously^30^,^38^. To test spontaneous pain behavior induced by bee venom, mice were placed on the surface of a 2 mm thick glass covered by a transparent Plexiglas box. The spontaneous pain was determined by counting the number of seconds the mice spent in lifting, licking and biting the injured hindpaw every 5 min interval for 1 h. To examine whether GAS exerts analgesic effects on bee venom-induced spontaneous nociception, GAS was administered intraperitoneally twice daily for 3 days before bee venom injection. Mechanical sensitivity was tested with manual application of Von Frey hairs to the plantar surface of hindpaw. Each filament was applied 10 times and the paw withdrawal response frequency (the percentage of positive responses to the stimulus) was recorded. The force of a particular filament required to elicit 50% frequency of paw withdrawal was expressed as the mechanical threshold^30^,^46^. Thermal sensitivity was tested by application of infrared heat to the plantar surface of hindpaw and the response latency was measured from an automated device readout, as described previously^30^,^46^ To avoid excessive tissue damage, the heat stimulus was cut off at 30 s. All the behavioral tests were conducted in a double-blinded manner. To evaluate the action of GAS on CFA-induced mechanical and thermal hypersensitivity, i.p. GAS or vehicle was administered twice daily for 5 days at 24 h after CFA injection when stable and significant hyperalgesia was produced.

#### Motor coordination testing

For testing motor function, we used a rotarod apparatus. After a training and acclimatization period, mice were placed on the rotating rod and the falling latency of each animal was recorded. The acceleration of rotating was from 2 to 60 r.p.m. over a 180 s period.

#### Intrathecal delivery of drugs *in vivo*

To enable intrathecal delivery at the level of lumbar spinal segments in mice, a polytetrafluoroethylene catheter was stereotactically inserted under anesthesia by 1% pentobarbital sodium. After a flush with 10 μl saline, the exterior end of catheter was sealed by heat. The mice were allowed to recover for 3 days. Any mouse showing motor deficits would be excluded. Penicillin antibiotics were used to prevent infection at the end of intrathecal catheterization. At 3 d after intrathecal catheterization, GAS (10, 50, 150 mM) was intrathecally applied in a volume of 5 μl followed by a 5 μl saline flush at 24 h after CFA inflammation.

### Immunohistochemistry labeling

Mice were subjected to hindpaw intraplantar injection with bee venom, killed and perfused transcardially with 4% paraformaldehyde at 2 h after bee venom. The spinal cord was removed, trimmed into several blocks and postfixed in the same fixative for 48 h, and then cryoprotected in 0.1 M PB containing 30% sucrose until the tissue block sank onto the bottom of the container. Vibratome sections (30 μm) of the spinal cord were immunostained for c-Fos protein with the avidin–biotin–peroxidase complex (ABC) methods. The sections of spinal cord were rinsed twice in 0.01 M PBS and then incubated with a solution containing 2.5% Triton X-100 and 3% bovine serum albumin (BSA) for 30 min at room temperature (22–25 °C). The sections were further incubated with a polyclonal antibody raised in rabbit against c-Fos (Cell signaling technology, 1:8000) for 24 h at 4 °C and then incubated with biotinylated goat anti-rabbit IgG (Vector, 1:200) and ABC complex (Vector, 1:200). The reaction product was visualized with 0.01% hydrogen peroxide and 0.05% diaminobenzidine in 0.05 M Tris–HCl buffer (pH 7.6). Between incubations the sections were rinsed three times in 0.01 M PBS, each for 10 min. There was no positive staining when PBS or normal rabbit serum was used instead of the antibody. The sections were then mounted on slides, dried, dehydrated, cleared and coverslipped. Immunoreactive cells in the superficial (lamina I-II) and deep layer (lamina IV–V) of spinal dorsal horn were microscopically counted in 8 sections per mouse from 5 mice per treatment group.

### Spinal cord slice patch-clamp recording

#### Spinal cord slice preparation

The methods used for obtaining a mouse spinal cord slice that retained an attached dorsal root and for patch-clamp recordings from spinal lamina I neurons have been described elsewhere^30^. In brief, mice were anaesthetized with urethane (1.2 g/kg, i.p.), and then lumbosacral laminectomy was performed at 24 h after CFA inflammation, at which remarkable mechanical and thermal hyperalgesia was stably produced. Transverse 350–450-μm-thick spinal cord slices with dorsal roots attached were obtained. The slices were stored in an incubation solution at room temperature (in mM: NaCl, 95; KCl, 1.8; KH_2_PO_4_, 1.2; CaCl_2_, 0.5; MgSO_4_, 7; NaHCO_3_, 26; glucose, 15; sucrose, 50; oxygenated with 95% O_2_, 5% CO_2_; pH 7.4). A slice was then transferred into a recording chamber and superfused with oxygenated recording solution at 3 ml min^−1^ at room temperature. The recording solution was identical to the incubation solution except for (in mM): NaCl 127, CaCl_2_ 2.4, MgSO_4_ 1.3 and sucrose 0.

#### Whole-cell patch-clamp recording

Spinal lamina I neurons were visualized with a 40X water-immersion objective using a microscope (BX51WI; Olympus) equipped with infrared differential interference contrast optics. Spinal lamina I neurons were found to lie within a distance of maximally 20 μm from the dorsal white/grey matter border. Standard whole-cell patch clamp recordings were performed with glass pipettes having a resistance of 4–6 MΩ in lamina I of spinal dorsal horn. The pipette solution consisted of (in mM): K-gluconate, 135; KCl, 5; CaCl_2_, 0.5; MgCl_2_, 2; EGTA, 5; HEPES, 5 and Mg-ATP, 5, pH 7.4 with KOH, measured osmolarity 300 mOsm. QX-314 (5 mM) was added to the pipette solution to prevent discharge of action potentials (APs). To measure excitatory postsynaptic currents (EPSCs) from neurons in lamina I, dorsal root was stimulated through a suction electrode with an isolated current stimulator and membrane potential was held at −70 mV. Test pulses of 0.1 ms with intensity of 3 mA were given at 30-sec intervals. Aδ-fiber or C-fiber evoked EPSCs (eEPSCs) were distinguished on the basis of the conduction velocity (CV) of afferent fibers (Aδ: 2–13 m/s; C: <0.8 m/s; calculated from the latency of EPSC from a stimulus artifact and the length of dorsal root), as described previously^47^. Aδ-fiber or C-fiber responses, respectively, were considered as monosynaptic in origin when the latency remained constant and there was no failure during stimulation at 20 Hz for 1 s, or when failures did not occur during repetitive stimulation at 2 Hz for 10 s^47^. Synaptic strength was quantified by the peak amplitudes of EPSCs.

#### Paired-pulse ratio and mEPSCs analysis

In a subset of experiments, paired-pulse stimuli with an inter-stimulus interval of 110 ms (0.1 ms pulse duration, 3 mA intensity, every 30 s) were applied to dorsal root. Paired-pulse ratio (facilitation or depression) of C-fiber-evoked EPSC was calculated as the amplitude of the second C-eEPSC divided by that of the first C-eEPSC in a pair. In a subset of experiments, miniature EPSCs (mEPSCs) were recorded at membrane potential of −70 mV in the presence of gabazine (10 μM), strychnine (1 μM), AP-5 (50 μM) together with TTX (0.5 μM) to block inhibitory synaptic transmission and NMDAR-mediated excitatory synaptic transmission.

#### Membrane properties analysis

For membrane properties analysis, membrane potential was held at −70 mV under current-clamp mode. Depolarizing current steps (500 ms in duration and 2 pA increments) were used to detect the AP. The AP threshold was determined by differentiating the AP waveform and setting a rising rate of 10 mV/ms as the AP inflection point. The AP amplitude was measured from the equipotential point of the threshold to the spike peak. The AP duration was measured at the half-width of the spike. Rise and decay slopes were detected from the AP threshold to the peak.

#### Drugs application

Drugs were applied by superfusion with a change in solutions in the recording chamber, being complete within 3 min. All drugs were first dissolved at 1000 times the concentration to be used, and then diluted to the desired concentration in ACSF solution immediately before use.

#### Data acquisition and analysis

Signals were gained using an Axopatch 700B amplifier (Axon Instruments, Foster City, CA, USA), low-pass-filtered at 5 kHz, and sampled at 10 kHz. Data were stored and analysed with a personal computer using the pCLAMP9.0 and Clampfit10.0 acquisition programmes (Axon Instruments).

### Statistical analysis

All results are presented as mean ± SEM. Student’s t-test, the Kolmogorov-Smirnov test or the analysis of variance (ANOVA) for random measures followed by post-hoc Fisher’s test or Dunnett’s test was used to determine statistically significant differences. *P* < 0.05 was considered to be statistically significant.

## Additional Information

**How to cite this article**: Xiao, M.-M. *et al*. Gastrodin protects against chronic inflammatory pain by inhibiting spinal synaptic potentiation. *Sci. Rep.* **6**, 37251; doi: 10.1038/srep37251 (2016).

**Publisher’s note:** Springer Nature remains neutral with regard to jurisdictional claims in published maps and institutional affiliations.

## Supplementary Material

Supplementary Information
