# Supplementary material for: Gastrodin protects against chronic inflammatory pain by inhibiting spinal synaptic potentiation
Source: Sci Rep. 2016 Nov 17;6:37251. doi: 10.1038/srep37251 (PMC5112517; doi:10.1038/srep37251)

**Gastrodin protects against chronic inflammatory pain by inhibiting spinal synaptic potentiation**

Mei-Mei Xiao^1,2,3,#^, Yu-Qi Zhang^1,2,#^, Wen-Ting Wang^2,#^, Wen-Juan Han^2,#^, Zhen Lin^4,#^, Rou-Gang Xie^2^, Zhi Cao^4^, Na Lu^2^, San-Jue Hu^2^, Sheng-Xi Wu^2,*^, Hui Dong^1,*^, Ceng Luo ^2,*^

^1^Department of Anesthesiology, Xijing Hospital, *Fourth Military Medical University, Xi’an, 710032, China*

^2^*Department of Neurobiology* *and Collaborative Innovation Center for Brain Science, Fourth Military Medical University, Xi’an, 710032, China*

^3^Department of Anesthesiology, Weifang People‘s Hospital, *151 Guangwen Road, Weifang, 261041, China*

*^4^Class 2013, School of Clinical Medicine, Fourth Military Medical University, Xi’an 710032, China*

^#^*These authors contributed equally to this work.*

^*^*Correspondence should be addressed to C.L. at luoceng@fmmu.edu.cn, or H.D. at* [*Dongh369@126.com*](mailto:Dongh369@126.com) *or S.X.W. at shengxi@fmmu.edu.cn*

Tel.: *+86 29 84779803*

Fax*: +86 29 83246270*

**Supplementary figure legends:**

Supplementary figure 1: (A) Four consecutive traces of mEPSCs in spinal lamina I neurons derived from control (left) and CFA-inflamed mice (right). (B) Quantitative summary showing that CFA inflammation induces a dramatic increase in the frequency of mEPSCs (left, n = 8 neurons from 4 mice, *P* < 0.05), but not in the amplitude (right, n = 8 neurons from 4 mice, *P* > 0.05) as compared to control mice.


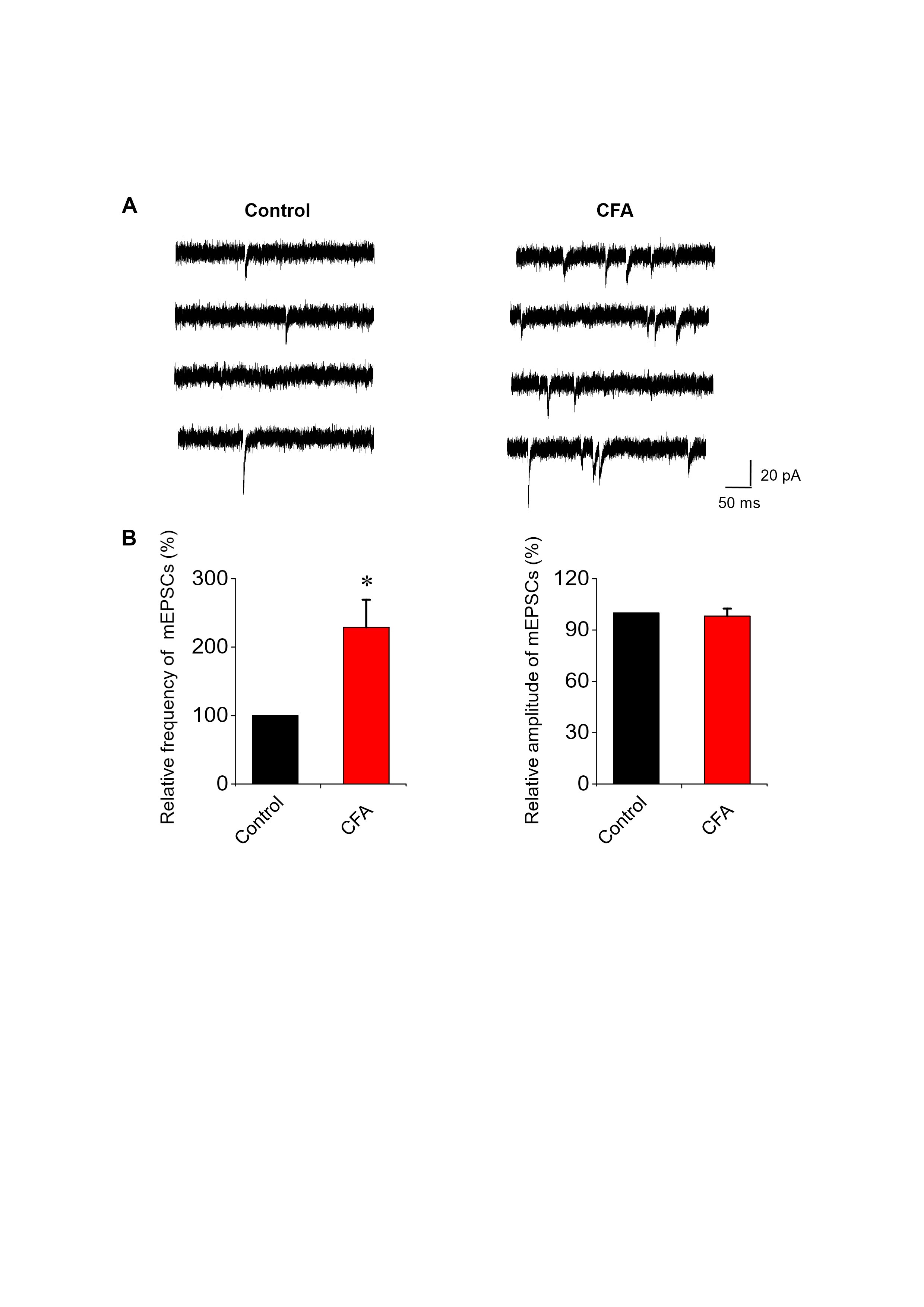

Supplement: Supplementary Information [file srep37251-s1.doc]
